# Sex Differences in Hepatic Inflammation, Lipid Metabolism, and Mitochondrial Function Following Early Lipopolysaccharide Exposure in Epileptic WAG/Rij Rats

**DOI:** 10.3390/antiox13080957

**Published:** 2024-08-07

**Authors:** Stefania Melini, Giovanna Trinchese, Adriano Lama, Fabiano Cimmino, Filomena Del Piano, Federica Comella, Nicola Opallo, Antonio Leo, Rita Citraro, Luigia Trabace, Giuseppina Mattace Raso, Claudio Pirozzi, Maria Pina Mollica, Rosaria Meli

**Affiliations:** 1 Department of Pharmacy, School of Medicine, University of Naples Federico II, 80131 Naples, Italy; stefania.melini@unina.it (S.M.); adriano.lama@unina.it (A.L.); federica.comella@unina.it (F.C.); nicola.opallo@unina.it (N.O.); mattace@unina.it (G.M.R.); meli@unina.it (R.M.); 2 Department of Biology, University of Naples Federico II, 80126 Naples, Italy; giovanna.trinchese@unina.it (G.T.); fabiano.cimmino@unina.it (F.C.); mpmollic@unina.it (M.P.M.); 3 Department of Veterinary Medicine and Animal Productions, University of Naples Federico II, 80137 Naples, Italy; filomena.delpiano@unina.it; 4 Science of Health Department, School of Medicine, University “Magna Graecia” of Catanzaro, 88100 Catanzaro, Italy; aleo@unicz.it (A.L.); citraro@unicz.it (R.C.); 5 Department of Clinical and Experimental Medicine, University of Foggia, 71122 Foggia, Italy; luigia.trabace@unifg.it

**Keywords:** genetic animal model, epilepsy, mitochondrial bioenergetics, oxidative damage, sex-dependent alterations, neonatal infections

## Abstract

Among the non-communicable neurological diseases, epilepsy is characterized by abnormal brain activity with several peripheral implications. The role of peripheral inflammation in the relationship between seizure development and nonalcoholic fatty liver disease based on sex difference remains still overlooked. Severe early-life infections lead to increased inflammation that can aggravate epilepsy and hepatic damage progression, both related to increased odds of hospitalization for epileptic patients with liver diseases. Here, we induced a post-natal-day 3 (PND3) infection by LPS (1 mg/kg, i.p.) to determine the hepatic damage in a genetic model of young epileptic WAG/Rij rats (PND45). We evaluated intra- and inter-gender differences in systemic and liver inflammation, hepatic lipid dysmetabolism, and oxidative damage related to mitochondrial functional impairment. First, epileptic rats exposed to LPS, regardless of gender, displayed increased serum hepatic enzymes and altered lipid profile. Endotoxin challenge triggered a more severe inflammatory and immune response in male epileptic rats, compared to females in both serum and liver, increasing pro-inflammatory cytokines and hepatic immune cell recruitment. Conversely, LPS-treated female rats showed significant alterations in systemic and hepatic lipid profiles and reduced mitochondrial fatty acid oxidation. The two different sex-dependent mechanisms of LPS-induced liver injury converge in increased ROS production and related mitochondrial oxidative damage in both sexes. Notably, a compensatory increase in antioxidant defense was evidenced only in female rats. Our study with a translational potential demonstrates, for the first time, that early post-natal infections in epileptic rats induced or worsened hepatic disorders in a sex-dependent manner, amplifying inflammation, lipid dysmetabolism, and mitochondrial impairment.

## 1. Introduction

The liver is a crucial organ in immunity and metabolism in children and adults [1]. However, how detrimental bacterial challenges in the post-natal period can influence liver adaptation and immune function has been overlooked. The hepatic immune response is involved in the systemic response to severe infection and is mainly orchestrated by Kupffer cells (KCs) [2,3]. These liver-resident macrophagic cells play a critical role in maintaining liver functions. They are responsible for the innate inflammatory and immune response, recruiting monocytes and neutrophils and stimulating T cells presenting antigens [4]. KCs engulf and eliminate pathogens and cell debris by phagocytosis and apoptotic cells and function as active sensors that detect immunoreactive fragments, including lipopolysaccharide (LPS). The latter is an endotoxin constituting a structural part of the outer membrane of Gram-negative bacteria, significantly stimulating the immune system [5], and is, therefore, widely applied in experimental models to resemble bacterial infections and sepsis [6]. Activated KCs secrete several mediators that regulate inflammation and homeostasis and drive the inflammatory response to liver injury [7]. Consistently, hepatocytes and immune cells cooperate in controlling systemic and local bacterial infections [7].

However, the mechanisms by which sepsis induces organ dysfunction and dysmetabolism have not been fully clarified. Mitochondrial alterations and reactive oxygen species (ROS) have been proposed in the pathogenesis of both neonatal and adult sepsis [8,9,10]. During sepsis, the liver plays two opposing roles: a source of inflammatory mediators and a target organ for a “boomerang effect” of the inflammatory mediators [6]. Consistently, neonatal rat endotoxemia is strictly linked to hepatic mitochondrial alterations [9].

**Scheme 1:**
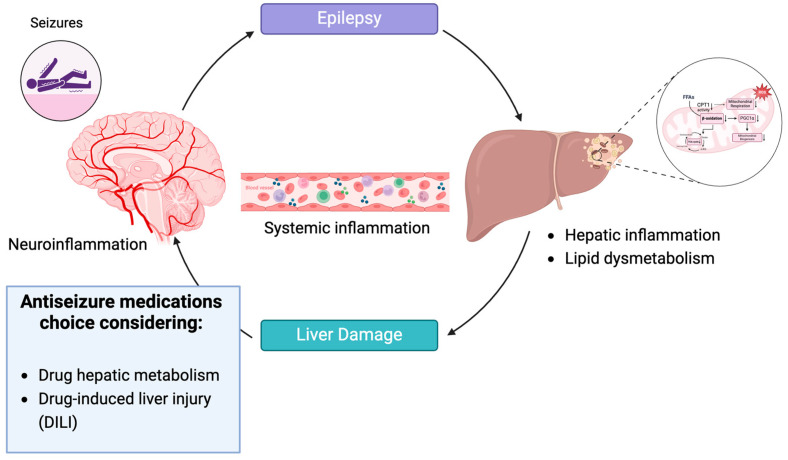
The relationship between epilepsy and liver damage.

Liver diseases, in both acute and chronic forms, can be associated with a wide spectrum of neurological manifestations, ranging in severity from subclinical changes to neurocritical conditions [11]. Among all, epilepsy is a non-communicable neurological disease characterized by abnormal brain activity with feasible peripheral implications. Indeed, the choice of the appropriate anti-epileptic therapy is crucial in patients with hepatic diseases to improve the treatment outcomes and reduce side effects in the liver (see Scheme 1).

In vivo studies have demonstrated that systemic and central nervous system inflammation is the main actor in the relationship between seizure predisposition and the development of nonalcoholic fatty liver disease (NAFLD) [12]. Indeed, severe early-life infections leading to sepsis may result in triggering hepatic and neuro-inflammation that can aggravate epilepsy. Furthermore, NAFLD has been recently identified as an independent risk factor for sepsis in a large clinical cohort, showing a strong relationship between mortality in NAFLD-associated sepsis and hepatic mitochondrial and energetic metabolism dysfunction [13]. 

Growing lines of evidence have heightened the importance of investigating sex-based differences in medicine and research about different pathologies, including infectious diseases [14,15]. Indeed, many bacterial infections show gender distinctions in pathophysiology, incidence, clinical presentation, disease course, and outcome [14].

Here, we examined the hepatic detrimental effect of an early LPS challenge in young epileptic rats (WAG/Rij), pointing out possible gender-related differences in liver inflammation and lipid metabolism alterations associated with mitochondrial oxidative damage. 

WAG/Rij rats, also named Wistar Albino Glaxo/Rijswijk rats, are a well-established animal model of human absence epilepsy. These animals share similarities with humans in brain activity recording and behavioral features, as well as the antiepileptic drug efficacy [16] and side effects, including hepatotoxicity [17]. However, the characterization of the metabolic profile of this useful strain and its sex differences have been still unexplored.

## 2. Materials and Methods

### 2.1. In Vivo Experimental Procedures and Ethics Statement

All the experiments were carried out in male and female Wistar Albino Glaxo/Rijswijk (WAG/Rij) rat pups obtained after mating male epileptic rats with dams. At post-natal day (PND) 3, WAG/Rij pups received the single intraperitoneal injection of lipopolysaccharide (LPS, 1 mg/kg, Sigma-Aldrich, Milan, Italy) to mimic early immune activation by gram-negative infection. At PND45, the animals of both sexes were sacrificed, and serum samples and fresh or frozen liver tissue were collected for the following biochemical and molecular determinations. Since the limitations were due to the impossibility of stereotaxically implanting 45-day-old rats for seizure quantification, preliminary results from electroencephalogram (EEG) recordings of 3-month-old male WAG/Rij rats challenged or not with LPS were obtained and reported in Supplementary Figure S1. All procedures involving animals comply with international and national law and policies, including the European Union (EU) Directive 2010/63/EU on animal experiments, animal research: reporting of in vivo experiments (ARRIVE) guidelines 2.0, https://www.arriveguidelines.org/resources (accessed on 22 September 2010), the Basel Declaration, and the national centre for the replacement, refinement and reduction of animals in research (NC3Rs) concept. The institutional committee on the ethics of animal experiments (CSV) of the University of Naples Federico II and the Italian Ministry of Health approved this procedure under protocol No. 591/2020-PR.

### 2.2. Biochemical Evaluations of Serum Hepatic Parameters, Lipid Profile, and Inflammatory/Immune Mediators

At PND45, blood was collected from all experimental groups. Then, serum was obtained by centrifugation at 2500 rpm at 4 °C for 12 min and stored at −80 °C for subsequent biochemical analyses. Serum parameters (cholesterol, triglycerides, alanine aminotransferase or ALT, aspartate aminotransferase or AST, alkaline phosphatase or ALP, and lactate dehydrogenase or LDH) were measured using commercially available ELISA kits. Moreover, the concentration of twenty-three pro- and anti-inflammatory mediators and factors of innate and adaptative immunity was determined using a high sensitivity kit (Bio-Techne; R&D Systems, Inc., Minneapolis, MN, USA) using the Bio-Plex System and Luminex xMAP technology (Bio-Rad Laboratories, Inc., Segrate, Milan, Italy). Cytokine concentrations were derived by interpolating the measured fluorescence intensities to standard curves and correcting for the corresponding dilution factor employed to achieve the minimum volume for analysis. Bio-Plex Manager 6.2 software was employed to calculate cytokine concentrations.

### 2.3. Mitochondrial Bioenergetics and Redox Status Evaluation

Hepatic mitochondrial isolation and oxidative capacities were performed as previously reported [17]. Oxygen consumption was polarographically measured using a Clark-type electrode in the presence of substrates and ADP (state 3) or with substrates alone (state 4). The high quality of mitochondrial preparations was indicated by high respiratory control ratio values in all groups, calculated as the ratio between states 3 and 4, according to Estabrook (1967). As previously reported [18,19], the degree of coupling was determined in the liver, according to the equation degree of coupling = √(1 − (Jo)sh/(Jo)unc. (Jo)sh represents the oxygen consumption rate in the presence of oligomycin that inhibits ATP synthase; (Jo)unc is the uncoupled rate of oxygen consumption induced by carbonyl cyanide p-trifluoromethoxy phenylhydrazone (FCCP), which dissipates the trans-mitochondrial proton gradient. (Jo)sh and (Jo)unc were measured using succinate (10 mmol/L) and rotenone (3.75 µmol/L) in the presence of oligomycin (2 µg/mL) or FCCP (1 µmol/L), respectively. The specific activity of the carnitine palmitoyltransferase (CPT) system and superoxide dismutase (SOD) was measured spectrophotometrically, as previously reported [20]. The rate of mitochondrial H_2_O_2_ release was assayed by measuring the linear increase in fluorescence caused by the oxidation of homovanillic acid in the presence of horseradish peroxidase. The protein content of the mitochondrial suspension was determined by the method of [21] using BSA as the protein standard. Furthermore, reduced glutathione (GSH) and oxidized glutathione (GSSG) concentrations in the liver were measured with the dithionitrobenzoic acid-GSSG reductase recycling assay [22]; the GSH-to-GSSG ratio was used as an oxidative stress marker.

### 2.4. ROS Assay

We diluted an equal volume of freshly prepared liver homogenate in 100 mM potassium phosphate buffer (pH 7.4), and then we added a final concentration of 5 μM dichloro-fluorescein diacetate (Sigma-Aldrich, Milan, Italy) in dimethyl sulfoxide for 15 min at 37 °C. A centrifugation at 12,500× *g* per 10 min at 4 °C was performed for the dye-loaded samples. Then, we mixed the pellet at ice-cold temperatures in 5 mL of 100 mM potassium phosphate buffer (pH 7.4) and incubated for 60 min at 37 °C. The HTS-7000 Plus-plate-reader spectrofluorometer (Perkin Elmer, Wellesley, MA, USA) was used to measure the fluorescence at 488 nm for excitation and 525 nm for emission wavelengths. ROS were quantified from the dichloro-fluorescein standard curve in dimethyl sulfoxide (0–1 mM).

### 2.5. RNA Extraction and Semi-Quantitative Real-Time (RT)-PCR

Total RNA isolated from the liver was extracted using TRIzol Reagent (Bio-Rad Laboratories, Hercules, CA, USA; 7326890) following the extraction kit’s protocol for RNA (NucleoSpin^®^, Macherey-Nagel GmbH & Co, Düren, Germany; FC140955N). cDNA was obtained using a High-Capacity cDNA Reverse Transcription Kit (Applied Biosystems, Foster City, CA, USA; 4374966) from 8 μg total RNA. RT-PCRs were performed with a Bio-Rad CFX96 Connect Real-time PCR System instrument and 2.0 software (Bio-Rad Laboratories, Inc., Segrate, Milan, Italy). The RT-PCR conditions were 15 min at 95 °C followed by 40 cycles of two-step PCR denaturation at 94 °C for 15 s, annealing extension at 55 °C for 30 s and extension at 72 °C for 30 s. Each sample contained 500 ng cDNA in 2X QuantiTect SYBR Green PCR Master Mix (204145) and primers pairs to amplify interleukin (IL)-1β (*IL1b*), cyclooxygenase-2 (*PTGS2*), tumor necrosis factor (TNF)-α (*TNF*), toll-like receptor 4 (*TLR4*), myeloid differentiation primary response gene (MyD)88 (*MYD88*), monocyte chemoattractant protein (MCP)1 (*CCL2*), peroxisome proliferator-activated receptor (PPAR)-α (*PPARa*) cluster of differentiation 36 (*CD36*), PPAR-γ (*PPARg*), PPAR-γ coactivator (PGC)-1α (*PPARGC1a*), ATP binding cassette subfamily G member 1 (*ABCG1*), and uncoupling protein 2 (*UCP2*), nuclear factor erythroid 2-related factor (NRF)2 (*NFE2L2*), NAD(P)H quinone dehydrogenase 1 (*NQO1*), heme oxygenase 1 (*HMOX1*) (Qiagen, Hilden, Germany), in a final volume of 50 μL. The relative amount of each studied mRNA was normalized to β-actin (*ACTB*) (Qiagen, Hilden, Germany) as a housekeeping gene, and data were analyzed according to the 2^−ΔΔCT^ method.

### 2.6. Statistical Analysis

All data shown are presented as mean value ± SEM. The Shapiro-Wilk normality test was performed to ensure the normal data distribution. Then, intragender and intergender comparisons were made using a two-way analysis of variance (ANOVA) followed by Bonferroni post hoc for multiple comparisons or a Student t-test, when appropriate. Differences among groups were considered significant at values of *p* < 0.05. Analyses were performed using GraphPad Prism 9 (GraphPad Software, San Diego, CA, USA).

## 3. Results

### 3.1. Gender Differences and LPS Effect on Biochemical Parameters and Inflammatory Mediators in Serum of WAG/Rij Rats

**Figure 1:**
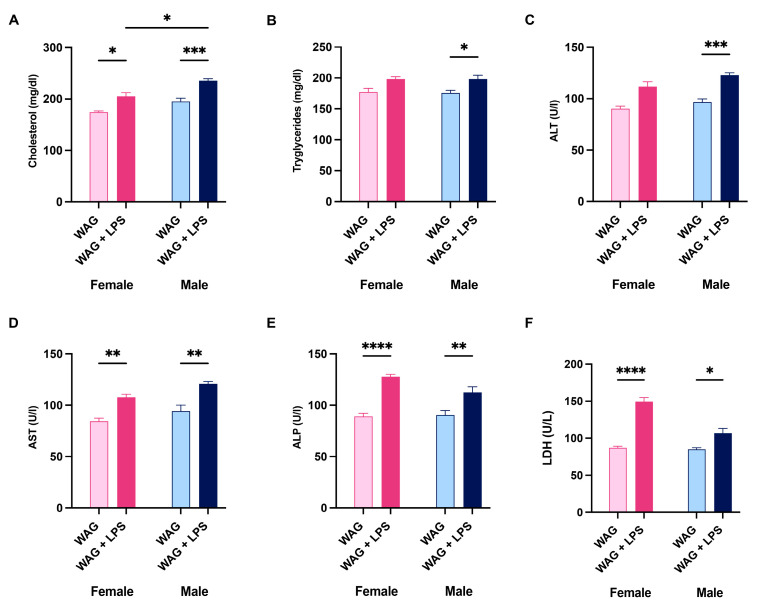
Sex differences in serum biochemical and hepatic parameters in WAG/Rij rats following LPS challenge. Serum (**A**) cholesterol and (**B**) triglycerides, (**C**,**D**) transaminases, (**E**) ALP, and (**F**) LDH were measured in all experimental groups (*n* = 6–7 animals each group) by ELISA assay. Data are presented as mean ± SEM. * *p* < 0.05, ** *p* < 0.01, *** *p* < 0.001, and **** *p* < 0.0001.

**Figure 2:**
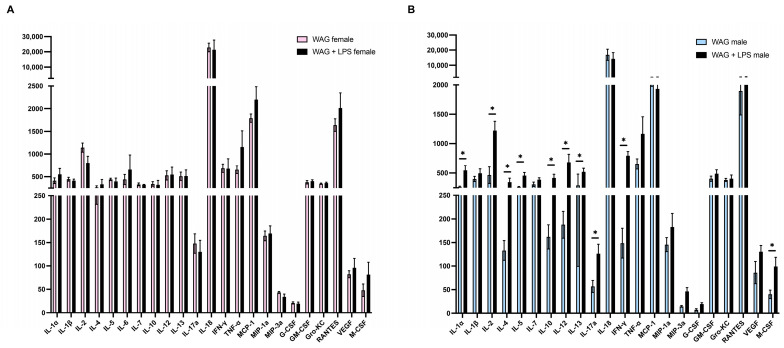
Intragender modulation of inflammatory and immune markers in the serum of epileptic rats following post-natal LPS injection. The evaluation of systemic levels of inflammatory and innate/adaptative immunity mediators was performed by Bio-Plex assay in (**A**) female and (**B**) male WAG/Rij rats, challenged or not with LPS, respectively (*n*= 6–7 animals in each group). Data are presented as mean ± SEM. * *p* < 0.05.

Intergender comparisons between male and female epileptic rats exposed to LPS at PND3 showed a similar increase in lipid serum profile, such as elevated cholesterol and triglycerides (Figure 1A,B), as well as higher levels of hepatic transaminases ALT and AST, ALP, and LDH (Figure 1C–F). No difference was observed between female and male WAG without LPS challenge in Figure 1. Notably, we showed a marked susceptibility of male epileptic rats rather than females to LPS-induced systemic inflammation, as proven by the higher serum levels of twelve out of twenty-three pro- and anti-inflammatory cytokines analyzed by BioPlex assay (Figure 2A,B). Specifically, LPS-challenged males showed increased serum concentrations of inflammatory and immune mediators (i.e., IL-1α, IL-12, and IL-13) and T cell-derived cytokines (i.e., IFN-γ, IL-2, IL-4, and IL-5). Other markers were also examined, such as IL-10, IL-17A, and M-CSF, which regulates the mobilization of neutrophils and macrophages/monocytes against bacterial infection. Their modulation in males suggested an attempt to counteract the damage caused by LPS. No intragender differences in female groups were found (Figure 2A). Intergender comparisons between male and female groups without LPS evidenced higher concentrations of some inflammatory cytokines and mediators of innate and adaptative immunity in females than in males (Supplementary Figure S2).

### 3.2. Hepatic Inflammation and Immune Response of Male and Female Epileptic Rats: Effect of an Early LPS Challenge

**Figure 3:**
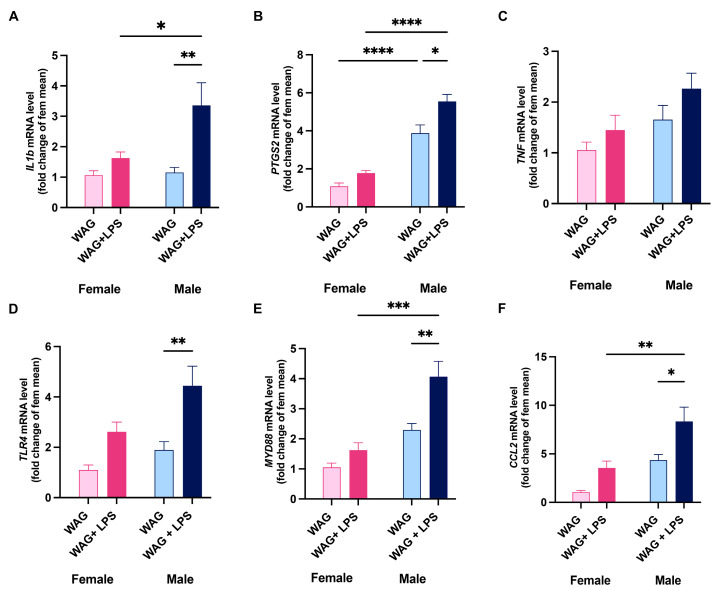
Gender-related differences in hepatic inflammation and immune response of WAG/Rij rats: effect of neonatal endotoxin infection. The mRNA expression of inflammatory (**A**) IL-1β, (**B**) COX-2, and (**C**) TNF-α, as well as (**D**) TLR4, (**E**) the coactivator MyD88, and (**F**) the chemoattractant MCP-1 was evaluated in the liver of all animals by Real-Time PCR (*n* = 6–7 animals each group). Data are presented as mean ± SEM. * *p* < 0.05, ** *p* < 0.01, *** *p* < 0.001, and **** *p* < 0.0001.

In the liver, we confirmed that male WAG/Rij rats were more prone to tissue inflammation and immune response to early LPS exposure compared with female animals. Indeed, we showed that the LPS challenge markedly increased the transcription of IL-1β, COX-2 (Figure 3A,B), as well as that of TLR4 and MyD88 (Figure 3D,E) and the monocyte chemoattractant protein (MCP)1 (Figure 3F). Notably, no statistical intergender and intragender difference was found in the mRNAs of TNF-α (Figure 3C).

### 3.3. LPS-Driven Metabolic Alterations in the Liver of Epileptic Rats: Sex-Related Differences

**Figure 4:**
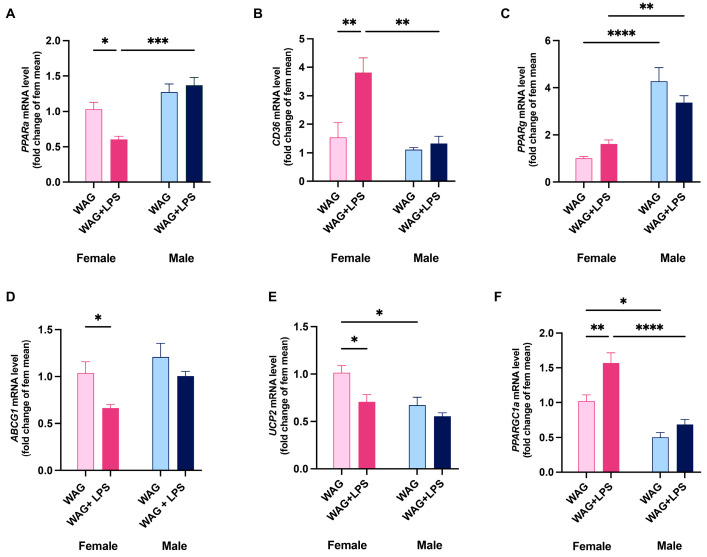
Hepatic metabolic changes of male and female epileptic rats after or not early LPS challenge. Sex-related and LPS-induced differences in lipid metabolism and mitochondrial damage were assessed by Real-Time PCR analysis. Indeed, the transcription of (**A**) PPAR-α and (**B**) CD36, as well as (**C**) PPAR-γ, (**D**) ABCG1, (**E**) UCP2, and (**F**) PGC1α, was evaluated in all experimental groups (*n* = 6–8 rats each group). Data are presented as mean ± SEM. * *p* < 0.05, ** *p* < 0.01, *** *p* < 0.001, and **** *p* < 0.0001.

Early endotoxin challenge induced a marked alteration in the hepatic lipid metabolism of female WAG/Rij rats, reducing the mRNAs of PPAR-α (Figure 4A), involved in the regulation of fatty acid oxidation (FAO) and increasing CD36 (Figure 4B), a key marker of steatosis. Moreover, LPS-insulted females showed a marked reduction of ABCG1 and UCP-2 transcription (Figure 4D,E), a mitochondrial ATP-dependent transporter and a liver-specific uncoupling protein, respectively. The mitochondrial damage was also suggested by the compensatory increase of PGC1-α (Figure 4F), a marker of mitochondrial biogenesis. No difference was found in PPAR-γ mRNAs in LPS-treated females vs. untreated ones (Figure 4C). Notably, intergender evaluations in unchallenged rats revealed marked differences in hepatic lipid metabolism. Indeed, we showed a significant increase in PPAR-γ expression (Figure 4C) and the reduction of PGC1-α (Figure 4D) and UCP2 (Figure 4F) in epileptic male rats rather than females.

### 3.4. Effect of LPS on Hepatic Mitochondrial Bioenergetics in Male and Female Epileptic Rats

**Figure 5:**
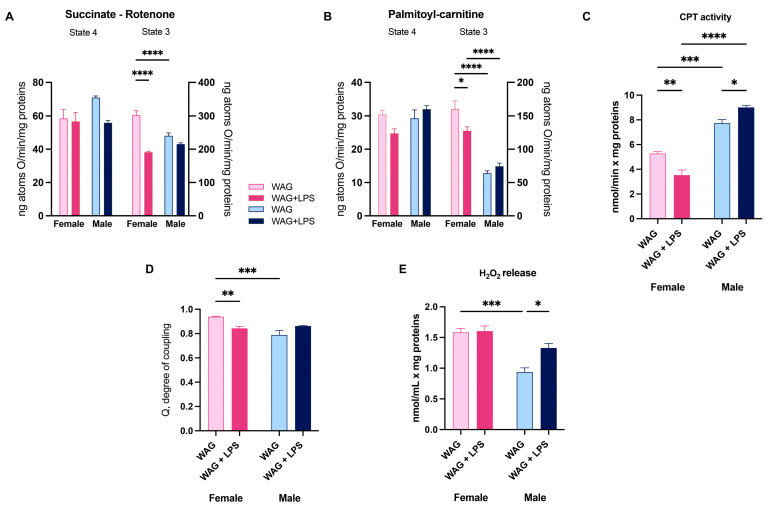
Sex-based differences in hepatic mitochondrial bioenergetics of WAG/Rij rats following post-natal endotoxin injection. Mitochondrial respiration in the presence or not of (**A**) succinate-rotenone and (**B**) palmitoyl carnitine as substrates was assessed in the liver of all animals by polarographic measurements (*n*= 5–6 animals in each group). (**C**) CPT activity, (**D**) the degree of coupling, and (**E**) H_2_O_2_ release into the isolated hepatic mitochondria of female and male WAG/Rij rats were measured by spectrofluorimetric analysis at PND45 (*n* = 6–7 animals in each group). Data are presented as mean ± SEM. * *p* < 0.05, ** *p* < 0.01, *** *p* < 0.001, and **** *p* < 0.0001.

LPS challenge induced a significant alteration of hepatic mitochondrial bioenergetics in females compared with males, as proven by the reduction of mitochondrial respiration in the presence of succinate-rotenone and palmitoyl carnitine as substrates (Figure 5A,B), and the decrease of β-oxidation displayed by the reduced mitochondrial CPT activity (Figure 5C), as the rate-limiting enzyme of FAO. A decrease in mitochondrial coupling was observed in female rats challenged with LPS (Figure 5D). Otherwise, in male epileptic rats, we noticed a strong release of hepatic mitochondrial H_2_O_2_ yield, while no differences were found in females (Figure 5E). LPS infection induced less mitochondrial damage in male rats, as shown by the slight decrease in mitochondrial function and no alteration in mitochondrial respiration in the presence of the specific substrates (Figure 5A,B) between WAG and WAG + LPS groups; otherwise, fatty acid oxidative capability by CPT activity was increased in male rats exposed to LPS.

### 3.5. Sex-Related Susceptibility to Oxidative Stress of WAG/Rij Rats: Effect of LPS Injection

**Figure 6:**
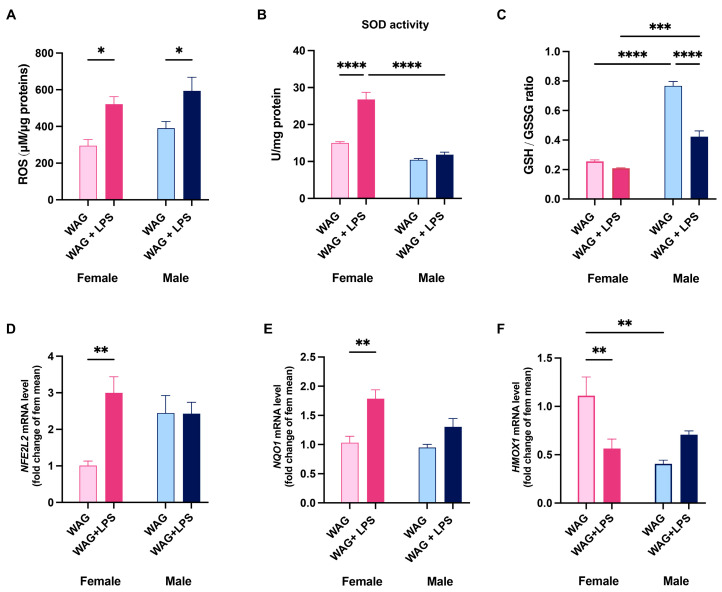
Hepatic oxidative stress and antioxidant defenses in epileptic rats: gender differences and effect of early LPS challenge. (**A**) ROS production, as well as (**B**) mitochondrial SOD activity, were spectrofluorimetrically measured in the liver of female and male WAG/Rij rats, challenged or not with LPS (*n*= 6–8 animals in each group). Moreover, (**C**) hepatic glutathione redox status (GSH/GSSG ratio) was calculated in all experimental groups (*n*= 5–6 animals in each group). The mRNA transcription of detoxifying factors (**D**) NRF-2, (**E**) NQO1, and (**F**) HO-1 was assessed by Real-Time PCR at PND45 (*n* = 6–7 animals in each group). Data are presented as mean ± SEM. * *p* < 0.05, ** *p* < 0.01, *** *p* < 0.001, and **** *p* < 0.0001.

Finally, we demonstrated that the LPS challenge induced oxidative damage in the livers of both genders increasing ROS production (Figure 6A). This LPS effect led to a gender-related activation of hepatic antioxidant defense, characterized by the increased mitochondrial SOD activity only in females (Figure 6B) and the altered hepatic glutathione redox status (GSH/GSSG ratio) in solely male WAG/Rij (Figure 6C). This latter finding was consistent with the increased H_2_O_2_ release, suggesting remarkable oxidative damage in challenged male rats. Notably, the LPS challenge also differently modulated the hepatic detoxifying system in female epileptic rats, increasing the gene expression of NRF-2 (Figure 6D) and its associated NQO1 (Figure 6E), while reducing HO-1 transcription (Figure 6F). No changes in the detoxifying system were revealed in male animals, injected or not with LPS (Figure 6D–F). However, in LPS-challenged females, the sustained antioxidant defense exerted by increased SOD activity and detoxifying factors cannot compensate for the increase in free radical production (Figure 6A).

## 4. Discussion

Neonatal sepsis is a serious life-threatening condition and a major cause of morbidity and mortality. Immunity and metabolism play pivotal roles in the host response to infection and the severe metabolic demands of early life [23]; indeed, it has been hypothesized that the defense strategies differentially employed between newborns and adults can be attributed to differences in systemic energy supply and demand, revealing at the cellular level as differences in immune-metabolic activity [24].

The liver, as a highly immunocompetent organ, can activate a developmentally regulated innate immune response to LPS-induced sepsis [25,26], with the associated induction of pro-inflammatory mediators and pathways occurring only after the first month of life [26]. These findings support the long-term hepatic implications of early life exposure to systemic inflammatory stress. Indeed, gut-derived bacterial endotoxins, including LPS, can contribute to the pathogenesis of NAFLD and steatohepatitis by activating KCs [27]. Notably, a link between mortality in NAFLD-associated sepsis and hepatic mitochondrial and energetic metabolism dysfunction has been shown [13].

Here, we demonstrated the detrimental effect of early LPS challenge in inducing liver damage in young WAG/Rij epileptic rats, establishing the gender-based differences in hepatic and systemic inflammation, immune response, lipid dysmetabolism, and associated mitochondrial oxidative damage. Contextually, these findings led us to characterize, for the first time, the gender metabolic profile of this useful strain regardless of early immunological damage. 

WAG/Rij rats are a valid animal model of epileptic absences appearing from the first month of age (30–45 days) and share many electroencephalographic and behavioral features with epilepsy in humans, including a comparable response to different antiepileptic drugs and related side effects, including hepatotoxicity [17]. 

Recent studies have shown a vicious circle between liver damage and epilepsy; in detail, how seizures can complicate the course of liver disease, and in turn, how liver damage can drastically reduce the therapeutic choice for epileptic patients. Indeed, antiepileptic drugs are commonly the cause of the so-called drug-induced liver injury (DILI). It has been recently reported a summary of all older antiseizure medications (ASMs) that induce liver damage as the primary side effect [28]. Among all, from a DILI network prospective study between 2004 and 2020 and the FDA adverse event reporting system (FAERS), lamotrigine, phenytoin, carbamazepine, valproate, levetiracetam, and diazepam had a “definite” or “highly likely” causality scores for DILI [28,29]. Contextually, we examined the difference between older and newer generation ASMs inducing DILI, surveying newly marketed ASMs for hepatotoxicity [29]. In this large post-marketing database, the majority of newer generation ASMs, including zonisamide, clonazepam, topiramate, gabapentin, ethosuximide, primidone, etc., were associated with reduced odds of DIL. Vigabatrin, tiagabine, and rufinamide reported zero evidence for DILI [29]. However, based on our knowledge, liver inflammation and lipid metabolism alterations were not examined concerning ASMs’ response in a sex-dependent manner. This issue can represent a limitation in translating our preclinical data to humans.

NAFLD is associated with a pro-inflammatory state and can induce peripheral and central inflammation, causing neurotoxicity and the induction of seizures [12,30]. 

In our experimental conditions, male and female epileptic rats, early exposed to LPS, exhibited a compromised hepatic function, as clearly shown by increased serum transaminases AST and ALT, and LDH, as well as an altered lipid profile. Otherwise, we evidenced a marked susceptibility of male WAG rats rather than females to systemic inflammation by LPS. In males, LPS-challenge increased serum levels of pro-inflammatory cytokines of innate immune response, T cell-derived cytokines, and other mediators produced in response to LPS-challenge that regulate the release of neutrophils and macrophages/monocytes against bacterial infection. Moreover, an increase of beneficial IL-10 and IL-17A was shown, suggesting the effort by the organism to counteract the damage caused by LPS. These results agree with other findings in animals and humans, where neonatal males appear more susceptible than females to bacterial infections [14]. Male mice show an inappropriate inflammatory response to sepsis and produce significantly higher levels of pro-inflammatory cytokines than females following endotoxemia or sepsis [31,32,33]. Previous data reported that in animal models, estrogen exerts a protective effect by maintaining adequate immune responses; conversely, ovariectomized rats are predisposed to sepsis, and the addition of estradiol restored immune function [34,35]. Concomitantly, progesterone can interfere with inflammatory and immune responses, inhibiting the release of proinflammatory mediators, increasing anti-inflammatory cytokines [36,37], and regulating T-cell activation [38]. Moreover, it has been shown that androgen receptor antagonism improves compromised immune functions and reduces mortality for sepsis in both preclinical and clinical studies [39]. In humans, the prevalence and severity of NAFLD are higher in men than in women during the reproductive age [40]. Indeed, after menopause, NAFLD occurs at a higher rate in women, suggesting that estrogen may have a protective role. Generally, animal models of NAFLD resemble sex differences observed in patients with more severe steatosis and steatohepatitis, with more pro-inflammatory/pro-fibrotic cytokines in males than females. In epileptic WAG/Rij rats, we confirmed the major predisposition of males than females to the inflammation and associated immune response primed by LPS at PND3 through the activation of COX-2, TLR4 pathway, and macrophage/monocyte recruitment in the liver at PND45. 

Notably, we found an intergender difference in epileptic rats without LPS, with the serum concentrations of some inflammatory cytokines and mediators of innate and adaptative immunity more elevated in females than males. A different systemic or tissue control of inflammation by extra sexual hormones in unchallenged epileptic animals cannot be excluded.

It has been reported that exposure to excess maternal fuels [41] or LPS [42] during the fetal period alters serum and hepatic lipid homeostasis, as well as liver morphology and mitochondrial health in adult mice and rat offspring, respectively. These detrimental changes promote oxidative stress and excess triglyceride storage, along with immune dysfunction, that drive the hepatic damage progression from NAFLD to steatohepatitis in adulthood [41]. 

Despite limited alterations of lipid profile in serum, early LPS infection led to marked lipid dysmetabolism in the liver of female WAG/Rij rats rather than males. Indeed, we showed the specific modulation of hepatic PPARs, which are differently involved in regulating lipid homeostasis. Moreover, in females, LPS increased the lipid transporter CD36, a crucial marker of steatosis responsible for the influx of fatty acids into the hepatocytes. PPARs are considered metabolic sensors and therapeutic targets in different liver diseases [43]. Previous studies have recognized the implication of PPAR-γ in the development and maintenance of steatosis in the liver [44,45,46] since its downregulation in hepatocytes avoids cellular lipid accumulation [47,48]. Otherwise, PPARα, abundantly expressed in the liver and regulated during inflammation in a gender-specific manner [49], acts as a lipid sensor responding to the influx of fatty acids by inducing the transcription of other genes encoding for mitochondrial, peroxisomal, and microsomal oxidation systems [50,51,52].

Interestingly, in the liver of LPS-insulted females, we found a reduced expression of mitochondrial ATP-dependent transporter ABCG1 and uncoupling protein UCP-2, index of mitochondrial damage, also suggested by the compensatory increase of PGC1-α, as an attempt to preserve or ensure an adequate mitochondrial biogenesis. These latter findings could explain the higher susceptibility of female rats to the detrimental effect of early LPS insult. However, we proved that epileptic male rats without challenge showed an increase in PPAR-γ expression and a reduced PGC1α and UCP2 alteration of hepatic lipid metabolism than females. 

Moreover, consistent with the alteration of lipid profile and metabolism, we strengthened a prominent hepatic mitochondrial damage in unchallenged male rats than females, revealing a major susceptibility to early immune infection and developing defects in mitochondrial bioenergetics in the liver. 

In female epileptic rats, the compromised mitochondrial respiration was evaluated in the presence of specific substrates acting on different complexes of the respiratory chain (succinate-rotenone and palmitoyl carnitine). The mitochondrial CPT activity was also shown and its reduction supported the limitation of FAO in LPS-insulted females. Notably, the two different gender-related mechanisms of LPS-induced liver injury (hepatic inflammation and lipid alterations) converge into oxidative damage in both sexes, confirmed by increased hepatic ROS. Female epileptic rats, exposed early to LPS, exhibited a reduction in mitochondria efficiency, indicated by a decreased degree of coupling. The electron transport and the ATP synthesis are closely coupled processes, but some of the energy generated by electron transport is uncoupled from ATP synthesis [53]. Instead, the reduction in mitochondrial efficiency allows the mitochondrial membrane potential to remain below the critical threshold for ROS production [54]. Indeed, uncoupling is a major mechanism for the adjustment of the membrane potential to control mitochondrial ROS emission. With the observed mild uncoupling in female animals, the mitochondria can avoid the excessive supply of electrons/reducing equivalents in the respiratory complexes and minimize the probability of electron interaction with oxygen [53]. Consistently, the increased oxidative stress triggered a remarkable compensatory rise in hepatic antioxidant defense by SOD, which is the first line of defense from oxidative stress [55] and detoxifying enzymes only in female WAG/Rij animals. The different modulation of NRF2 pathway intermediates, such as NQO-1 and HO-1, suggest that the effect of an early immune challenge can negatively modulate the hepatic oxidative balance during epilepsy. Any improvement in the cellular redox state (GSH/GSSG) in the liver of female rats was shown.

It is known that LPS-induced sepsis and related inflammatory or stress conditions raise seizure susceptibility [56]. In this context, it should be considered the relevant role of oxidative stress and redox dysregulation in epileptic patients independently by antiepileptic pharmacological therapy [57]. Specifically, the increased levels of oxidative biomarkers, including malondialdehyde, protein carbonylation, and nitric oxide, have been found in the brain and peripheral tissues of both human patients and epileptic animals, with any sex differences [57,58,59]. In the vicious relationship between epilepsy and oxidative damage, mitochondrial dysfunction plays a pivotal role, participating in the immunoinflammatory response [60,61]. It has been shown that many individuals with epilepsy exhibit concomitant mitochondrial disorders [62]. Furthermore, mitochondrial SOD2 knockdown in mice causes a remarkable rise in developing spontaneous motor seizures [63]. Moreover, the impairment of hepatic oxidative balance relieved mainly in male rats is confirmed by the altered GSH/GSSG ratio. The increase in GSH usually occurs in response to oxidative stress, and a decrease in GSH can worsen disease [64]. Reduced GSH levels are also observed in several liver diseases [65] and in diseases associated with inflammation caused by microbial infections [66,67].

## 5. Conclusions

In summary, our data show, for the first time, a characterization of the metabolic profile at the systemic and hepatic levels of WAG/Rij epileptic strain; contextually, we describe with a high translational potential that an early post-natal infection can predispose epileptic animals to develop or exacerbate hepatic disorders enhancing the different sex-related susceptibility. Female WAG/Rij has physiologically higher serum levels of many inflammatory mediators, and this difference was not evidenced in hepatic tissue and parameters, as well as a lipid profile. Conversely, lipid metabolism was similar between female and male WAG without the LPS challenge. However, LPS significantly impacts lipid metabolism in female rats. Regarding mitochondria bioenergetics, a deep alteration in male rats, insulted or not with LPS, was shown, even if post-natal infection also reduced mitochondrial respiration and fatty acid oxidation in females. Despite the observed gender-related differences, our findings suggested that the effect of an early immune challenge can negatively modulate the hepatic oxidative balance during epilepsy in both genders, as demonstrated by the increase of ROS production.

Contemplating and then targeting the co-morbidity between liver damage and epilepsy can represent a fascinating health challenge also in the optimal therapeutic choice aimed at limiting the course of these pathologies, both characterized by a strong immune component.

## Data Availability

The data presented in this study are available on request from the corresponding author.

## Supplementary Materials

The following supporting information can be downloaded at https://www.mdpi.com/article/10.3390/antiox13080957/s1, Figure S1: Intergender evaluation of serum inflammatory/immune markers in WAG/Rij rats; Figure S2: Effects of LPS administration on (A) the number and (B) duration of characteristic SWDs in 3-month-old WAG/Rij rats. References [68,69] are cited in the Supplementary Materials.
