# Supplementary material for: Sex Differences in Hepatic Inflammation, Lipid Metabolism, and Mitochondrial Function Following Early Lipopolysaccharide Exposure in Epileptic WAG/Rij Rats
Source: Antioxidants (Basel). 2024 Aug 7;13(8):957. doi: 10.3390/antiox13080957 (PMC11351225; doi:10.3390/antiox13080957)
Supplement: Supplementary file 1 [file antioxidants-13-00957-s001.zip › antioxidants-3113565-supplementary.pdf]

Supplementary Materials

1. Supplementary Methods

1.1 Biochemical evaluations of serum inflammatory/immune mediators

At PND45, blood was collected from all experimental groups. Then, serum was obtained by centrifugation at 2500 rpm at 4 °C for 12 min and stored at -80°C for subsequent biochemical analyses. The concentration of twenty-three out of pro- and anti-inflammatory mediators, and factors of innate and adaptative immunity were obtained by a high sensitivity kit (Bio-Techne; R&D Systems, Inc., USA) using the Bio-Plex System and Luminex xMAP technology (Bio-Rad Laboratories, Inc., USA). Cytokine concentrations were derived by interpolating the measured fluorescence intensities to standard curves and correcting by the corresponding dilution factor employed to achieve the minimum volume for analysis. Bio-Plex Manager software was employed to calculate cytokine concentrations.

1.2 Electroencephalogram (EEG) recordings of 3-month-old male WAG/Rij rats

Male WAG/Rij rat pups (n = 12) were randomly allocated into two subgroups; the first subgroup (n = 6) was intraperitoneally (i.p.) treated with LPS (from E. coli, serotype O55:B05, Sigma-Aldrich, MO) (1 mg/kg body weight) at three days of age (PND3). Vehicle rats received the same volume of sterile saline solution (100 µl). After the end of treatment, WAG/Rij rats were normally housed up to the age of ~3 months. Subsequently, LPS-treated and untreated WAG/Rij rats, under general anesthesia [mixture of tiletamine/zolazepam; 1:1; Zoletil 100®, 50 mg/kg i.p. (VIRBAC Srl, Milan, Italy)], were stereotaxically implanted with 3 cortical electrodes for EEG recordings attached to a 3-channel rat head-mount (8239-SE3; Pinnacle Technology, Stoke-on-Trent, UK) for seizure quantification, as previously described [1]. Quantification of epileptic seizures was based on the number and the duration of EEG spike-wave discharges (SWDs) according to well-established criteria. Briefly, the number and duration of SWDs (nSWDs and dSWDs) for each rat were summarized in 30-min intervals (epochs) and scored by visual inspection of the EEG recordings; all recordings were analyzed by two independent researchers that were blinded to the treatment [2].

1.3 Statistical analysis

All statistical procedures were carried out using GraphPad Prism 10.0 software (GraphPad Software Inc., La Jolla, CA, USA). To assess LPS effects on absence seizures parameters, EEG recordings were subdivided into 30 min epochs, and both total and single duration and number of SWDs were assessed separately for every epoch. Such values were averaged, and data acquired were expressed as mean ± S.E.M. All tests used were two-sided, and $p \leq 0.05$ was considered significant.

2. Supplementary Results

2.1 Intergender differences on inflammatory mediators in serum of WAG/Rij rats

The intergender evaluation in epileptic rats without LPS by Bio-Plex assay revealed more elevated serum concentrations of T cell-derived cytokines, such as interleukin (IL)-2, IL-4, IL-5, and interferon (IFN)-γ, a mediator of innate immune activation such as IL-12, as well as macrophage inflammatory protein-3 (MIP3A) as a markers of macrophages/monocytes

recruitment (Figure S1); these findings indicated a pronounced inflammatory state in female rats which could justify the less susceptibility to LPS challenge of females than males.

2.2 Early LPS accelerates the onset of seizures in young adult male WAG/Rij rats

Preliminary results revealed no significant increase of nSWDs in LPS-challenged male WAG/Rij rats compared to age-matched control (vehicle) rats, while dSWDs were worsened by LPS injection in male epileptic rats. In detail, EEG recording analysis obtained from vehicle 3-month-old WAG/Rij rats (n = 6) showed a mean number of SWDs (nSWDs) of 4.91 ± 0.63, with a mean total duration (dSWDs) of 22.52 ± 6.06 s and a mean single duration (sSWD) of 4.24 ± 0.38 for a 30-min epoch. In contrast, acute treatment with LPS in WAG/Rij rats (n = 6) produced an increase of the mean nSWDs and dSWDs by ~ 14% (p = 0.25) and 39% (p = 0.04), respectively (Figure S2A-B). The mean sSWD value did not significantly differ between LPS-treated and untreated WAG/Rij rats, at this time point considered.

Supplementary Figures

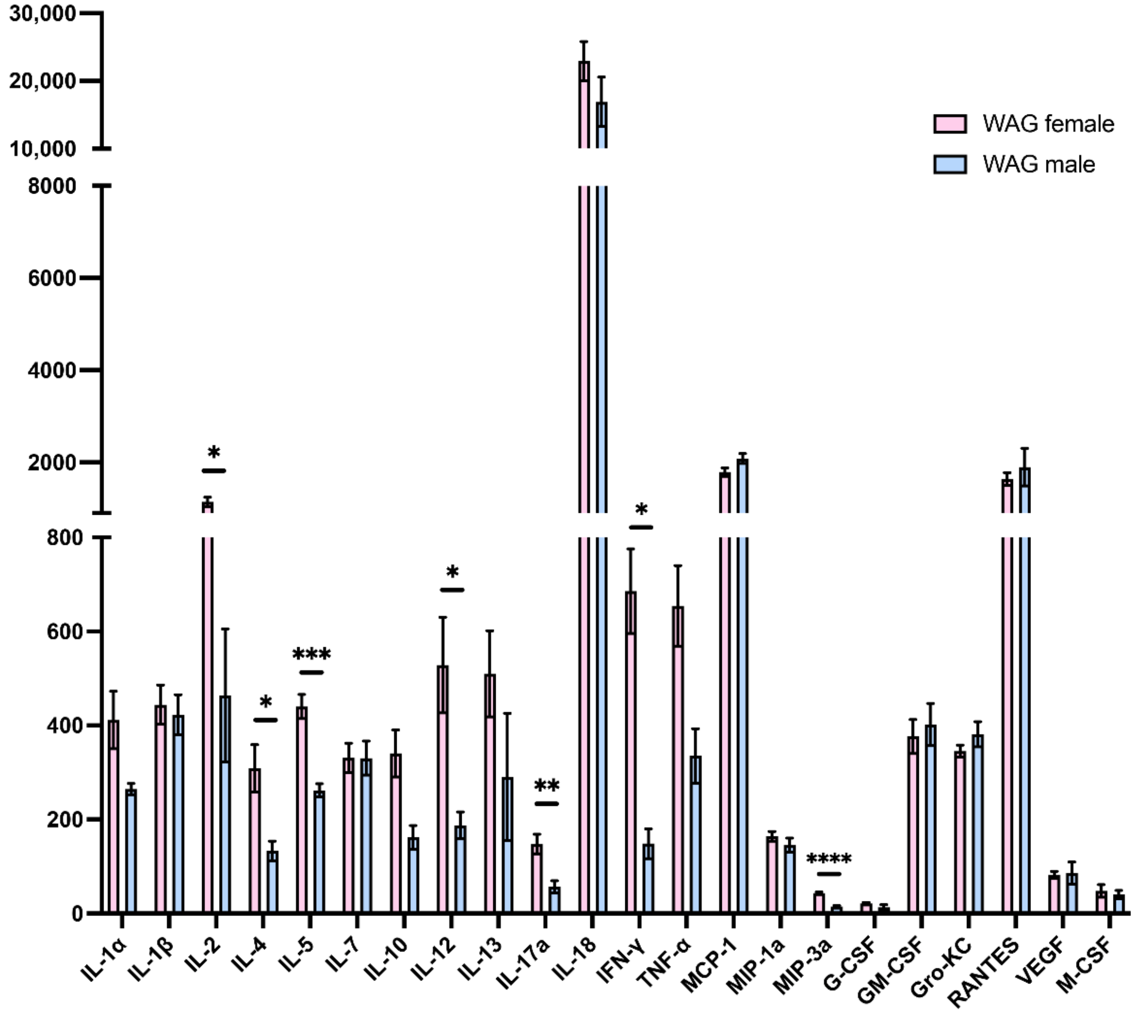

Figure S1. Intergender evaluation of serum inflammatory/immune markers in WAG/Rij rats. Serum concentrations of inflammatory and immune markers were measured in male and female WAG/Rij rats by Bio-Plex System assay (n= 5–6 rats each group). Data are presented as mean ± SEM. * $p < 0.05$, ** $p < 0.01$, *** $p < 0.001$, and **** $p < 0.0001$.

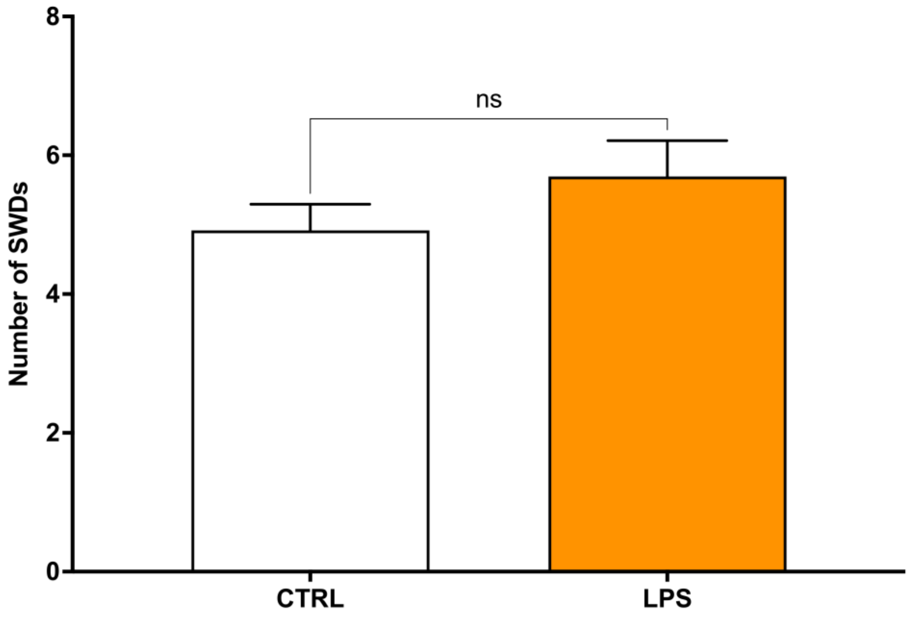

(**A**)

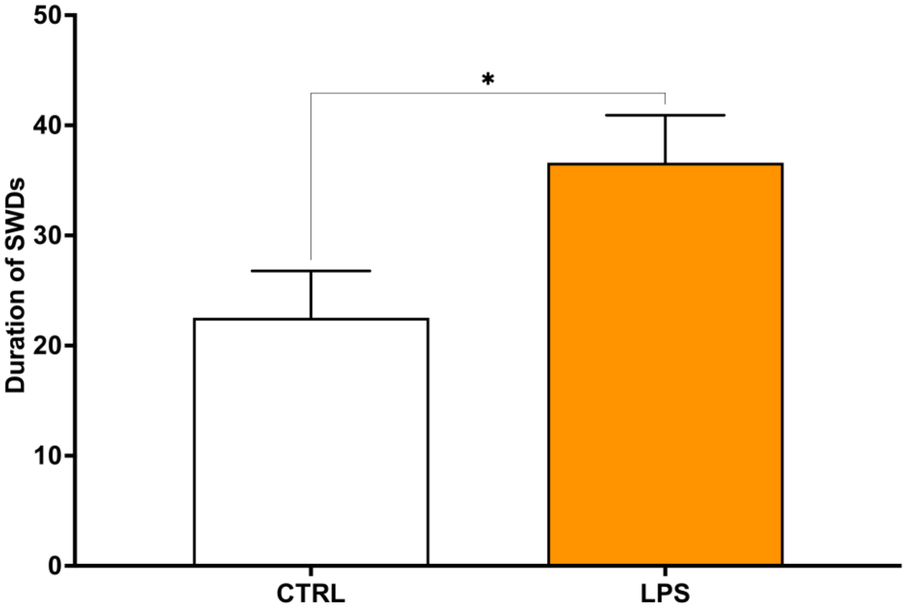

(**B**)

Figure S2. Effects of LPS administration on (**A**) the number and (**B**) duration of characteristic SWDs in 3-month-old WAG/Rij rats ($n = 6$ per group). Data values are means ± SEM for every 30 min epoch. * Significantly different ($p \leq 0.05$) from age-matched untreated control rats. Data are presented as mean ± SEM. * $p < 0.05$. CTRL = control; LPS = lipopolysaccharide; nSWDs = number of spike-wave discharges; dSWDs = duration of spike-wave discharges. ns: not significant.

References

1. Leo, A.; Nesci, V.; Tallarico, M.; Amodio, N.; Cantafio, E.M.G.; De Sarro, G.; Constanti, A.; Russo, E.; Citraro, R. IL-6 Receptor Blockade by Tocilizumab Has Anti-absence and Anti-epileptogenic Effects in the WAG/Rij Rat Model of Absence Epilepsy. *Neurotherapeutics* **2020**, *17*, 2004–2014.
2. Tallarico, M.; Leo, A.; Guarnieri, L.; Zito, M.C.; De Caro, C.; Nicoletti, F.; Russo, E.; Constanti, A.; De Sarro, G.; Citraro, R. N-acetylcysteine aggravates seizures while improving depressive-like and cognitive im-pairment comorbidities in the WAG/Rij rat model of absence epilepsy. *Mol. Neurobiol.* **2022**, *59*, 2702–2714.
